# Retraction: The impact of agricultural insurance on farmers’ income: Guangdong Province (China) as an example

**DOI:** 10.1371/journal.pone.0327331

**Published:** 2025-07-02

**Authors:** The PLOS One Editors

After this article [1] was published, PLOS noted concerns about compliance with the Authorship policy and about the following reporting and reference issues:

- The data sources and specific datasets used in this study are not described in sufficient detail to ascertain how the results or conclusions were derived, or allow others to reproduce the study.
- Numerous statements in the Introduction are not supported by data or by published and cited work.
- We identified errors in 4 references (7, 13, 18, 23) and have been unable to verify 23 references (1, 2, 3, 19, 21, 28–38, 40, 44, 45–48, 55). Also, 24 of the 56 works cited were published at least 10 years before [1] was submitted.
- The article cited as Reference 56 was retracted after [1] was published.

The corresponding author stated that some references are outdated, no longer available to the public, or were translated from Chinese. They were unable to provide specific information about the dataset or references by the deadline provided.

The *PLOS One* Editors retract this article due to the unresolved concerns which question the reliability of this article.

MAB and MD did not agree with the retraction. ZX and ZZ either did not respond directly or could not be reached.
